# Contributions to Obstetric Jurisprudence

**Published:** 1859-05

**Authors:** Horatio R. Storer

**Affiliations:** Boston


ART. I.—*Contributions to Obstetric Jurisprudence.* By HORATIO R. STORER, M.D., of Boston.

### CRIMINAL ABORTION (CONTINUED.)

### III. ITS VICTIMS.

WE have hitherto discussed abortion in the abstract, as a crime, and in its relations to the community at large. We have seen its heinous nature, and its awful extent.*

* Since our last article, the report of the Committee appointed in 1858 to investigate the Health Department of the City of New York has appeared, and we find that our statements regarding the frequency of the crime in the metropolis are fully cor-

The division of the subject now to be examined naturally presents itself under a fourfold aspect, numerical, relational, social, and medical; representing respectively the multitude of the victims of abortion, their character as parent and child, their standing in the community, and the degree, whether unto death or otherwise, of their suffering.

So far as statistics will at present allow, the numerical relations of the victims of abortion have already been fully discussed, the yearly thousands of fœtal and the frequent maternal deaths; one sacrifice at least, that of the child, being implied in every case.

Various incidental questions suggest themselves in this connection, curious, and by some, though erroneously, thought to be of judicial importance. Two of them will be here adverted to; they are the age of the mother and that of the fœtus.

It was formerly supposed, as in infanticide, that criminal abortion was seldom resorted to save for the concealment of shame; and as seductions, confessedly frequent in comparison with adultery, are most common in youth, it was laid down as probable that wilful abortions are most frequent between the years corresponding, from sixteen to twenty-five. Subsequent experience, however, has disproved this conclusion, and it is evident that the only real limit to its later occurrence is the menstrual climacteric. In many instances of marriage, abortions are resorted to at once, in others after the family has reached a certain point, and are thence regularly continued.

The age of the fœtus is of much more interest; not, as will ultimately appear, for the same reason as in cases of infanticide, but as proving to a certain extent one of the facts we have already considered, the error prevalent regarding the commencement of fœtal life.

It was Orfila's opinion that criminal abortion was most frequent in the two first months of pregnancy. This would naturally have been supposed the case, as then some doubt might always obtain regarding its existence, and the excuse that the measures resorted to were for the purpose of preventing ill effect from an abnormal menstrual suppression would be more

---

roborated. Not merely are additional official statistics on this point given (pp. 182, 183), but valuable testimony from Drs. Griscom (pp. 25, 30), McNulty (p. 55), Francis (p. 64), and Bulkley (p. 133). Dr. Reese's paper on Infant Mortality, republished by the Committee (pp. 90–100), from the Transactions of the American Medical Association for 1857, also contains incidental reference to the frequency of abortion, and for its direct and earnest dealing with the subject deserves unqualified commendation.

In this connection we would call attention to the evidence of the extent of the crime in Boston, afforded since our own remarks upon that point were in type, by Dr. Walter Channing. (Boston Med. and Surg. Journal, March 17, 1859.)

available. Devergie, on the other hand, was inclined to put the limits of greatest frequency at from three months to four and a half; while Briand and Chaudé thought the crime more common in the third month than the fifth, and in this last month much more frequent than even in the first or second. Tardieu also came to a similar conclusion.* He ascertained, that of 34 cases investigated by himself, 25 were in from the third to the sixth month, most in the third; 5 in the two first months; 4 in the seventh and eighth; or that the cases in the third month, or shortly after, were five times as numerous as at either an earlier or a later period, and nearly three times as numerous as in both combined.

Upon examining the Register of the Morgue, we find its statistics strikingly corroborative of these deductions. We have already seen that from 1837–54 there had been deposited at the Morgue 692 fœtuses of less than nine months. Of these,

| | | | |
|---|---|---|---|
| 23 | were from the | first to the second month; |
| 79 | " | " | second to the third; |
| 108 | " | " | third to the fourth; |
| 158 | " | " | fourth to the fifth; |
| 150 | " | " | fifth to the sixth; |
| 97 | " | " | sixth to the seventh; |
| 48 | " | " | seventh to the eighth; |
| 29 | " | " | eighth to the ninth. |

It has been stated that 519, or five-sixths of them all, were not over six months, and it now appears that on a scale twenty times larger than that given by Tardieu from his own experience, nearly two-thirds of the fœtal deaths induced by abortion were in from the third to the sixth month of pregnancy; the three periods included giving a much larger proportion than any others, and the two last of them being almost identical. The extreme paucity shown by the above table in the first and ninth months, and the decrease in the seventh and eighth from those preceding, are worthy of remark. It is probable that the sudden increase may be attributed to mental reaction after the first shock occasioned by the absolute certainty of pregnancy is past; and the subsequent decrease to the fact that in many attempted criminal abortions during the later months children are born alive, the mother's courage then proving insufficient for infanticide and its greater and more probable punishment.

The truth seems to be that while frequently the crime is committed in the very earliest period of pregnancy, in the belief that possibly all alarm may be false, in most cases the woman waits a month or two to be sure that she really is pregnant. After the sixth month the comparative rarity

---

* Annales d'Hygiène Publique, 1856, p. 122.

of the crime is undoubtedly owing to the fact that fœtal life is then rendered certain by the perceived movements of the child, the more strongly as pregnancy advances.

It was formerly thought that the induction of criminal abortion was confined to the unmarried alone; there exists, however, abundant proof to the contrary. Had such been the fact it would be reasonable to suppose that with the increase of marriages, the number of premature and still-births would have lessened. In proportion, however, as the number of marriages has increased, so has the number of fœtal deaths, and in similar ratio the number of living births has declined; proving, as Husson was compelled to acknowledge of Paris, "a decrease in the fecundity of legitimate unions,"* explainable in nowise but by a criminal cause. Direct statistics on this point are still wanting, and must come, almost entirely, from medical men. The writer's published experience has been already referred to; subsequent practice has only confirmed it.

Again, the victims of abortion are not confined to the inhabitants of cities. Of the prosecutions in France during 1851–53, it appears that but little more than a tenth of the whole number occurred in the Department of Seine.† But we have already furnished much stronger evidence on this point.

Finally, though the excessive extravagance of the day, entailing as it does to so many an annual expenditure incommensurate with their income, is accountable in no small degree for the crime, yet it cannot always be alleged. There is too much reason to believe that with our communities it is as with others. In France it appears that the poor and uneducated have on the whole the largest families, contrary in some respects to established hygienic rules; while, on the other hand, the rich are inclined as far as possible to restrict the number of their children, often indeed stipulating beforehand, at marriage or previously, that such shall be few or none.‡

But not only must we believe that this crime prevails in our midst to an almost incredible extent among the wealthy and educated and married; we are compelled to admit that Christianity itself, or at least Protestantism, has failed to check the increase of criminal abortion. It is not astonishing to find that the crime was known in ancient times, as shown by evidence previously given, nor that it exists at the present day among savage tribes, excused by ignorance and superstition; but that Christian communities should especially be found to tolerate and to practice it, does almost exceed belief.

---

* Les Consummations, etc. † Comptes Rendus Annuels, etc.

‡ MAYER, Des Rapports Conjugaux, considérés sous le triple point de vue de la population, de la santé et de la morale publique. Paris, 1857.

In the previous remarks, the French have been compared with our own people, not merely because of their published statistics, but because from their loose morals and habits of life they might be thought to represent the extreme to which the crime would be likely to obtain among civilized nations. We have shown reason to suppose that we equal them in guilt, if we do not exceed them. Now it must be borne in mind that the French are Catholic; that the rules of that church do not, as has been generally supposed, permit abortion in the earlier months,* but that they rigidly exercise over fœtal life throughout pregnancy the most absolute guard and supervision;† instances of this in cases where craniotomy is supposed to be indicated, are familiar to every practitioner. The church, here omnipotent against the physician, and desiring that every created human being should receive the benefit of baptism, demands that fœtal death must have taken place previously to the operation proposed. It does not seek for the signs of fœtal life, but takes this for granted and requires that its absence must be proved before allowing the removal of the child, even though this is necessary to save the mother's life.

The rule runs thus: "Sedulam operam dent sacerdotes, ut quantum poterunt, impediant nefandum illud scelus quo adhibitis chirurgicis instrumentis infans in utero interficitur. Omnis fœtus quocumque tempore gestationis editus baptizetur, vel absolute, si constet de vitâ; vel sub conditione, nisi evidenter pateat eum vitâ carere."‡

In cases where instrumental delivery is absolutely necessary, the ceremony of baptism as ordinarily administered is of course impossible. But as this is deemed so important by the Catholic Church,§ even to the sacrifice of the mother, any means of overcoming this obstacle, fatal to countless maternal lives, should be accounted, if found practicable, of the utmost importance. For this purpose, therefore, I do not hesitate to recommend intra-uterine baptism, the consecrated element being carried to the child, if too high to be reached by the hand, by a sponge and staff, or if necessary, by a syringe, as was long ago sanctioned by the theologists of the

---

* CANGIAMILA, Embryologia Sacra, p. 15.

† In verification of this statement I am enabled to quote from the last authorized edition of the Canon Laws of the Church of Rome. "Omnes, qui abortûs seu fœtûs immaturi, tam animati quam inanimati, formati vel informis, ejectionem procuraverint, pœnas propositas et inflictas tam divino quam humano jure, ac tam per canonicas sanctiones et apostolicas constitutiones quam civilia jura adversus veros homicidas incurrere, hâc nostrâ perpetuo valiturâ constitutione statuimus et ordinamus." REIFFENSTUELL, Jus Canonicum Universum, tome iii. Paris, 1854.

‡ Decreta Synodi plenariæ Episcoporum Hiberniæ, apud Thurles habitæ anno 1850. Art. de Baptismo, p. 20.

§ Dublin Review, April, 1858, p. 100.

Sorbonne;* in the absence of a clergyman, if the case is urgent, the rite accounted so sacred being performed by the physician in attendance rather than to allow the death of the mother. I know that many of the profession, from fear of ridicule or dislike to sanction what they do not believe in, would shrink from such a duty. Is it not, however, due to humanity in every way to prevent this frequent murder? for such, by our apathy and neglect, the mother's death in these cases becomes. Can we, as Christians, refuse *any* aid? I am not ashamed to acknowledge that for myself, though no Catholic, I have performed this intra-uterine baptism, where delivery without mutilation was impossible. I could neither conscientiously assert the child's death, nor allow the mother to linger till it should occur, while the friends, in the absence of a priest, whose presence it was impossible to procure for many hours, would not permit the operation. Remonstrance was useless; had I refused I should have been morally responsible for the almost inevitable death of the woman; it was, therefore, simply my duty.

I shall now quote from an admirable letter I have lately received from the Catholic Bishop of Boston. The extracts are couched in so forcible language, and their spirit is so thoroughly Christian, that I need not apologize for their length, or that they recapitulate arguments I have already advanced.

"The doctrine of the Catholic Church," remarks Bishop Fitzpatrick, "her canons, her pontifical constitutions, her theologians without exception teach, and constantly have taught, that the destruction of the human

---

* DEVENTER, 1734, p. 366; STERNE, Tristram Shandy, p. 54; Med. Times and Gazette, Aug., 1858, p. 196. Though the fact of this decision has been doubted, it is nevertheless strictly true. Through the kindness of Bishop Fitzpatrick I have been favored with a copy of BARRY's Medico-Christian Embryology, as presenting upon this point the authorized and generally received doctrine of the Catholic Church. I quote the following from the chapter "On Baptism in Impracticable and Difficult Labors:"

"In case of impacted head and at all times that one is obliged to apply the forceps, whether at one of the straits or in the pelvic excavation, it becomes necessary to baptize the child on the part which presents at the uterine orifice after the rupture of the bag containing the waters.

"In order to baptize the child, a syringe charged with natural water may be used. If this be not at hand, a person may use a sponge, or a linen or cotton rag, wetted with water, which is to be carried to the child by the fingers, a pair of forceps, or any other suitable contrivanee, and then squeezed or pressed on the surface of the part presenting." (Loc. cit., p. 45.)

"Any person, whether man, woman or child, may baptize an infant when in danger of death." (Ibid., p. 76.)

If the facts now stated should be generally known and acted upon by the profession, hundreds of lives, infant and maternal, would annually be saved.

fœtus in the womb of the mother, *at any period from the first instant of conception*, is a heinous crime, equal, at least, in guilt to that of murder. We find it distinctly condemned as such even as far back as the time of Tertullian, (at the end of the second century,) who calls it *festinatio homicidii*, a hastening of murder. The Pope, Sixtus the Fifth, in a bull published in 1588, subjects those guilty of the crime to all the penalties, civil and ecclesiastical, inflicted on murderers. It is denounced and reprobated in many other canons of the church.

"The reason of this doctrine (apart from the authority of the church) must, it seems to me, appear evident upon a little reflection. The very instant conception has taken place, there lies the vital germ of a man. True, it is hidden in the darkness of the womb, and it is helpless; but it has sacred rights, founded in God's law, so much the more to be respected because it is helpless. It may be already a living man, for neither mothers nor physicians can tell when life is infused; they can only tell when its presence is manifested, and there is a wide difference between these two things. At any rate, it is from the first moment potentially and *in radice* a man, with a body and a soul destined most surely by the will of the Creator and by his law, to be developed into the fullness of human existence. No one can prevent that development without resisting and annulling one of the most sacred and important laws established by the Divine Author of the universe, and he is a criminal, a murderer, who deals an exterminating blow to that incipient man, and drives back into nothingness a being to whom God designed to give a living body and an immortal soul.

"From this it follows that the young woman whose virtue has proved an insufficient guardian to her honor, when she seeks by abortion to save in the eyes of man that honor she has forfeited, incurs the additional and deeper guilt of murder in the eyes of God, the judge of the living and the dead. Who can express what follows with regard to those women, who, finding themselves lawfully mothers, prefer to devastate with poison or with steel their wombs, rather than bear the discomforts attached to the privilege of maternity, rather than forego the gaieties of a winter's balls, parties, and plays, or the pleasures of a summer's trips and amusements?

"But abortion," the Bishop continues, "besides being a direct crime against a positive law of God, is also an indirect crime against society. Admit its practice, and you throw open a way for the most unbridled licentiousness, you make woman a mere instrument for beastly lust. Every woman is somebody's mother or daughter or sister or wife; or she bears all these relations at once. Whatever protection, therefore, we would claim for a woman because she stands in any of these relations to us, we should also extend to all women, because they bear some one of all these relations to others. Most assuredly, then, we should remove none of the safeguards that protect female virtue. But if we take away the responsibility of maternity, we destroy one of its strongest bulwarks.

"It affords me pleasure," he concludes, "to learn that the American Medical Association has turned its attention to the prevention of criminal abortion, a sin so directly opposed to the first laws of nature, and to the designs of God, our Creator, that it cannot fail to draw down a curse upon the land where it is generally practiced."*

Such being the doctrine of the Catholic Church, and statistics nevertheless proving that its so stringent statutes against the destruction of fœtal life, enforced alike by denouncement and excommunication, are constantly broken in Catholic France, it is more than probable, from the existence of this fact, in addition to all the various causes of the crime we have seen equally prevailing there and in this country, that the actual number of abortions is much greater with ourselves; at least with the American and native portion of our population. We have indeed shown that this is really the case in Massachusetts.†

Viewing this subject in a medical light, we find that death, however frequent, is by no means the most common or the worst result of the attempts at criminal abortion. This statement applies not to the mother alone, but, in a degree, to the child.

We shall perceive that many of the measures resorted to are by no means certain of success, often indeed decidedly inefficacious in causing the immediate expulsion of the fœtus from the womb; though almost always producing more or less severe local or general injury to the mother, and often, directly or by sympathy, to the child.

The membranes or placenta may be but partially detached, and the ovum may be retained. This does not necessarily occasion degeneration, as into a mole or hydatids, or entire arrest of development. The latter may be partial, as under many forms, from some cause or another, does constantly occur; if from an unsuccessful attempt at abortion, would this be confessed, or indeed always suggest itself to the mother's own mind? Fractures of the fœtal limbs prior to birth are often reported, unattributable in any way to the funis, which may amputate indeed, but seldom

---

* MS. Letter, dated Nov. 14th, 1858.

† It is not of course intended to imply that Protestantism, as such, in any way encourages, or indeed permits, the practice of inducing abortion; its tenets are uncompromisingly hostile to all crime. So great, however, is the popular ignorance regarding this offence, that an abstract morality is here comparatively powerless; and there can be no doubt that the Romish ordinance, flanked on the one hand by the confessional, and by denouncement and excommunication on the other, has saved to the world thousands of infant lives.

break a limb. A fall, or a blow, is recollected; perhaps it was accidental, perhaps not, for resort to these for criminal purposes is very common. In precisely the same manner may injury be occasioned to the nervous system of the fœtus, as in a hydrocephalic case long under the writer's own observation, where the cause and effect were plainly evident. Intra-uterine convulsions have been reported; as induced by external violence they are probably not uncommon, and the disease thus begun may eventuate in epilepsy, paralysis, or idiocy.

To the mother there may happen correspondingly frequent and serious results. Not alone death, immediate or subsequent, may occur, from metritis, hemorrhage, peritonitic or phlebitic inflammation, from almost every cause possibly attending not merely labor at the full period, comparatively safe, but miscarriage,—increased and multiplied by ignorance, by wounds and violence; but if life still remain, it is too often rendered worse than death.

The results of abortion from natural causes, as obstetric disease, separate or in common, of mother, fœtus or membranes, or from a morbid habit consequent on its repetition, are much worse than those following the average of labors at the full period. If the abortion be from accident, from external violence, mental shock, great constitutional disturbance from disease or poison, or even necessarily induced by the skilful physician in early pregnancy, the risks are worse. But if, taking into account the patient's constitution, her previous health and the period of gestation, the abortion has been criminal, these risks are infinitely increased.* Those who escape them are few.

In thirty-four cases of criminal abortion reported by Tardieu, where the history was known, twenty-two were followed, as a consequence, by death, and only twelve were not. In fifteen cases necessarily induced by physicians, not one was fatal.†

It is a mistake to suppose, with Devergie, that death must be immediate, and owing only to the causes just mentioned. The rapidity of death, even where directly the consequence, greatly varies; though generally taking place almost at once if there be hemorrhage, it may be delayed even for hours where there has been great laceration of the uterus, its surrounding tissues, and even of the intestines;‡ if metro-peritonitis ensue, the patient may survive for from one to four days, even indeed to seven and ten. But there are other fatal cases, where on autopsy there is revealed no appreciable lesion; death, the penalty of unwarrantably interfering with

---

* PASSOT, Des dangers de l'avortement provoqué dans un but criminel; Gazette Méd. de Lyon, 1853.

† Ann. d'Hygiène, 1856, p. 147.

‡ DUBOIS and DEVERGIE, ibid., tome xix. p. 425; tome xxxix. p. 157.

nature, being occasioned by syncope, by excess of pain, or by moral shock from the thought of the crime.*

That abortions, even when criminally induced, may sometimes be safely borne by the system, is of little avail to disprove the evidence of numberless cases to the contrary. We have instanced death. Pelvic cellulitis, on the other hand; fistulæ, vesical, uterine, or between the organs alluded to; adhesions of the os or vagina, rendering liable subsequent rupture of the womb, during labor or from retained menses, or, in the latter case, discharge of the secretion through a Fallopian tube and consequent peritonitis; diseases and degenerations, inflammatory or malignant, of both uterus and ovary; of this long and fearful list, each, too frequently incurable, may be the direct and evident consequence, to one patient or another, of an intentional and unjustifiable abortion.

We have seen that in some instances the thought of the crime, coming upon the mind at a time when the physical system is weak and prostrated, is sufficient to occasion death. The same tremendous idea, so laden with the consciousness of guilt against God, humanity, and even mere natural instinct, is undoubtedly able, where not affecting life, to produce insanity. This it may do either by its first and sudden occurrence to the mind; or, subsequently, by those long and unavailing regrets, that remorse, if conscience exist, is sure to bring. Were we wrong in considering death the preferable alternative?

### IV. ITS PROOFS.

It is by no means an easy thing in all cases to obtain evidence that an abortion has occurred; still more difficult, that it has been intentionally induced. As most laws read, it is necessary at the outset to prove the existence of pregnancy; as many still stand, it must be shown that the woman has quickened. These requisitions are unwise and unjust, and under them, if insisted on by adroit counsel, it is almost useless to pursue prosecution. In the earlier months, before quickening has occurred or the fœtal pulsations have become evident to the ear, it is impossible, as I have elsewhere insisted,† ever to be sure of the existence of pregnancy, and yet attempts at its termination are then in no degree less criminal. The only infallible sign of pregnancy is the sound of the fœtal heart, not always to be detected, even by the double stethoscope.

Putting aside, therefore, the question of the existence of pregnancy and of fœtal life, as taken for granted on the one hand by the attempt

---

* Ibid.

† Review of MONTGOMERY's Signs of Pregnancy. This journal, March, 1857, p. 249.

at their termination, and as proved on the other by this result, it is found that the evidence of abortion classifies itself into proofs of its occurrence, of its commission, of the criminal intent, and of the identity of the party accused.

1. *The Occurrence.*

The abortion may perhaps be known to have taken place by confession or witness; in either case requiring no further demonstration. Instances are not rare, however, where suspicion merely may exist, and the fact must be proved by collateral testimony; and this in two cases, where the woman is still alive, and where she is dead.

The general history of the case, even if pregnancy and delivery be equally denied, may throw some light on its true nature. This as given, no matter how affected by the evidence of interested or implicated witnesses, may be probable or improbable; as in an instance related by Burns,* where her sudden lessening in bulk was ascribed by a patient to a night's profuse sweating, of course an impossible result. But, on the other hand, care is necessary lest from its rarity of occurrence or its improbability, the reality should be disbelieved. Cases are on record where innocent women, suffering from retention of menses or from ovarian disease, and suddenly relieved by a critical and spontaneous discharge, have on suspicion of abortion lost character and even their lives. If the statements contradict each other, this fact of itself may reveal the truth.

There are at times difficulties in proving delivery at the full period of pregnancy, as is well known. The earlier in gestation, if the patient survive, the more these difficulties are enhanced. The occurrence of normal labor cannot be discovered with any certainty by a personal examination after eight days have elapsed,† those of an early abortion not even after only one or two.‡ The signs of delivery that are well marked at the full period, the general symptoms then obtaining, the size of the uterus, ascertained by the hand and sound, lacerations of the perineum and cervix, the lochia, the state of the breasts, abdomen, vagina, and vulva, all of little value except in conjunction with each other, are proportionately less defined as we go back, until near the commencement of pregnancy it becomes impossible to distinguish the abortion from severe hemorrhage or from menorrhagia, unless by detecting the impregnated ovum.

If the patient is dead, and too long time have not elapsed since the

* Principles of Midwifery, p. 547.

† BAUDELOCQUE, tome i. p. 115; FODERE, ii. p. 17; MARC, Dict. de Méd., i. p. 228; MONTGOMERY, Signs of Pregnancy, p. 578; DEVERGIE, Méd. Légale, i. p. 244.

‡ RYAN, p. 267; TARDIEU, loc. cit.

supposed occurrence, decision is often more easy; many facts in the case being generally known, and concealment being less possible. If the ovum, but partially detached, be still retained, the fact is self-evident; if it has been discharged, and concealed or lost, there will still be present the recent corpus luteum and other well-known signs, in proportion to the period of the pregnancy. Allowance, in this latter respect, must be made for the possibility of partial uterine contraction after death, as is sometimes known to occur at the full period;* the writer has seen it to a marked degree some time previous to the expiration of pregnancy, at a Cæsarean section after death from laryngitis, occurring in the Edinburgh Maternity Hospital.

### 2. *The Commission.*

Allowing the fact of the occurrence of an abortion to be proved or granted, it becomes necessary to discover its cause, whether accidental, natural, or intentional. Of this it will be found that the proofs are both positive and negative; drawn from the history of the case and from personal examination of the patient and the fœtus. The value of each of these elements is increased in proportion as it is compared with the other; but "this I wish most especially to have noted, that WHEREVER THERE IS A MISCARRIAGE, THERE IS ALWAYS PRESENT SOME ACTUAL, PERCEPTIBLE, AND OFTEN TANGIBLE CAUSE."†

The story of the patient may be to one effect, and that of other parties involved, to a very different one; if the first is corroborated by the second, it may again, as has already been remarked, present or not the likelihood of truth. If the habit of aborting at a certain period has existed, which of course cannot be alleged in a first pregnancy, if the patient has had sudden fright or grief, or is known to have been accidentally injured, the chances are to be considered in her favor, in the absence of proof to the contrary. The converse of this statement, however, must not be considered as always and necessarily true. Women have time and again suffered shipwreck, undergone torture, been thrown from a height, and otherwise severely injured, and yet have escaped miscarriage; while, on the other hand, they may repeatedly have aborted before, and yet passing safely their usually critical point, may without trouble go on to the full period. In still other cases there may exist local disease, pelvic or uterine, which, if left alone, would of itself occasion miscarriage.

The character of the abortion is not without its value, whether occurring

---

* CLARKE, Trans. of Soc. for Impr. of Med.-Chir. Knowledge, iii. p. 290; BAUDELOCQUE, i. p. 123, note; LEROUX, Traité des Pertes, Obs. xiii. p. 25 MONTGOMERY, loc. cit., p. 618.

† GARDNER, of New York, note to TYLER SMITH'S Lectures on Obstetrics, p. 203.

suddenly and without apparent cause, or preceded by maternal disease or the signs of fœtal death.

Examination of the mother, though proof that she has herself been injured is not necessary to establish the crime, may reveal local wounds and mutilations; or their absence, which, however, by no means goes to prove that violence may not have been inflicted; and, on the other hand, as in a case lately reported by the writer to the Boston Society for Medical Observation, traces of former violence in instrumental or other labors may remain, and to such an extent as to give to the touch every character of a recent and criminal interference.* The fœtus may show pre-existing and natural disease sufficient to account for the effect apparent, or may present the signs of direct and intentional interference. Recent scars of venesection on the arms and feet, and of leech-bites, especially on the upper and inner parts of the thighs, are suspicious in a patient who has aborted, unless they were evidently required by the state of her previous health. If signs of irritant injections into the vagina are present, they are ground for more than suspicion.

The instrument, where used, with which the operation has been performed, may sometimes be identified; though this is almost impossible unless by confession or direct testimony. The weapons resorted to by the unprofessional are various; knitting kneedles, pen handles, skewers, goose quills, pieces of whale-bone, and even curtain rods, are among the number. The finger alone, except where the uterus is prolapsed or can be depressed, and the os is very soft and patulous, is seldom if ever sufficient for the deed. If a physician be accused, it is important to notice with what instrument the crime is said to have been performed, if before witnesses, and whether it was introduced openly or under pretence of a digital examination.

The sensations of the patient at the time, are also in different cases unlike each other. In some instances nothing unusual is observed, in others a prick or probing, but in most an acute and tearing uterine pain, often followed by syncope or an hysterical attack. Slight but immediate hemorrhage generally occurs, save in professional cases, increased by compelled exercise, prolonged baths or ergot.

The time ensuing before the expulsion of the fœtus is an element not

---

* Am. Journ. of the Med. Sciences, April, 1859. In the instance referred to, the cervix had been deeply and extensively lacerated, forceps having been used in four previous labors; while depressions existing between the old cicatrices and half filled and ragged with clots, were decidedly suggestive of punctured wounds. The true nature of the case was rendered evident by its past history, and corroborated by the fact that the patient was a Catholic; the latter being a point to which I am inclined to attach much importance, for reasons already given.

to be lost sight of. In 34 cases reported by Orfila, the minimum observed was $13\frac{1}{2}$ hours, the maximum 6 days; in 36 cases by Tardieu, the minimum was 5 hours, the maximum 11 days. Of these last cases, however, 29 were within 4 days.

It cannot be alleged in excuse that the sex of the child, so fatal in advanced pregnancy, has any influence in producing early abortion. In 293 premature still-births reported by Collins,* 146 were male and 147 female, bearing the proportion of 100 to 100. Nor can the plea of Drs. Gordon Smith, Good, Paris, and Copeland, that as a fœtus born before the seventh month has a slender chance of surviving, its murder should be viewed with leniency,† be allowed. Such arguments, that the perils and dangers to which the fœtus is naturally subjected should lessen the criminality of attempts at its destruction, are without foundation, and when advanced by physicians are utterly unworthy the profession.

### 3. *The Intent.*

We shall hereafter discuss the perpetrators of the crime, and the emergencies which can alone justify the induction of premature labor or obstetric abortion. We shall see that by none save medical men can such necessity ever be known; it is, therefore, apparent that the intent may frequently be judged from the relation of the parties implicated, and the excuses offered by them. It will also often appear from the other circumstances of the case. That the child was likely to be born a bastard, and to be chargeable to the reputed father, would be evidence to that effect; and proof of the clandestine manner in which the drugs were procured or administered would tend the same way.‡

On the part of the mother, bastardy also, the having denied the existence of pregnancy, concealed its expelled product, expressed an intention or desire to abort, made a known application for this purpose, visited a notorious abortionist, taken alleged specifics, or given similar advice to a friend, are all presumptive evidence; as are also the having neglected to send for aid when needed, or refused to take precautions or remedies when prescribed. In like manner, evidence of criminal intent would seem apparent, if drugs generally supposed abortive had been advised or given to a pregnant woman, or violence of any kind usually productive of the effect in question, even to tooth-drawing, had been hastily or unnecessarily used.

Here, as in many other cases where no malice is expressed or openly indicated, the law will imply it; if, for instance, a man wilfully poisons

* Practical Treatise, p. 275. † RYAN, Med. Jurisprudence, p. 282.

‡ ROSCOE, Law of Evidence, 242.

another, in such a deliberate act the law presumes malice, though no particular enmity can be proved.* Malice is not confined in its legal definition to ill-will toward one or more individual persons, but is intended to denote an action flowing from any wicked or corrupt motive, a thing done *malo animo*, where the fact has been attended with such circumstances as carry in them the plain indications of a heart regardless of social duty and fatally bent on mischief; and, therefore, it is implied from any deliberate or cruel act against another.† The rule is, that the implication of malice arises in every such case, and all the circumstances of accident, necessity, or infirmity are to be satisfactorily established by the party charged, unless they arise out of the evidence and attending circumstances; if they do not, there is nothing to rebut the natural presumption of malice. This rule is founded on the plain and obvious principle that a person must be presumed to intend to do that which he voluntarily and wilfully does in fact do, and that he must intend all the natural, probable, and usual consequences of his own act.‡

The standing in society of the accused, unless notoriously bad, should of course be allowed to weigh but little; the less the likelihood of the crime, the greater, from example and previous education, its guilt.

If violent purging or vomiting have been resorted to without any apparent reason, or to a greater extent than ordinarily prescribed or required; or if leeches have been applied to the thighs, to the number of an hundred or more, as instanced by Tardieu, or the like, there is certainly ground for strong suspicion. And here it is that the criminal liability of careless or ignorant physicians becomes evident. In cases such as we have referred to, it would be very difficult for a successful defence to be offered, providing the pregnancy had been suspected by those not implicated, were the statutes on abortion properly drawn and enforced.

It has been ruled, and very justly, that attempts at the crime, though unsuccessful; or effective, yet the ovum retained as mole, hydatids, skeleton, mummy, or putrilage; and whether the woman be pregnant or not, and if pregnant, whether the child be alive, dead, or abnormally developed or degenerated, should be amenable as though fully consummated.§ We have seen the frequent difficulty in proving fœtal life; the attempt at its destruction shows the belief in its existence, and the intent. The proofs will here of course be of a different nature. The signs of delivery will be absent, and all evidence from the product of conception, unless the mother's death ensue; in which event, as in the other fatal cases

* ARCHBOLD, Crim. Pleading, 491; 1 HALE, 455.

† DAVIS, Crim. Justice, 482.

‡ Ibid., 483.

§ Reg. *v.* Haynes; Reg. *v.* Goodall; Rex *v.* Phillips.

we have considered, and on the principle just laid down, procedure might be had on the charge either of abortion or homicide; but it must not be forgotten, as we early pointed out, that immediate death from the shock may occur, and no lesion of any kind be found. The patient or parties interested are proved by the attempt to have supposed pregnancy existing, and to have behaved as though this were the fact.

The age of the patient is not of consequence; nor is that of the fœtus, save as corresponding with the alleged period of pregnancy, in case any doubt exist as to its own identity or that of the mother, and as bearing on the statement we have already attempted to prove, that criminal abortion is comparatively rare after the period of quickening, and, therefore, on the probability of intent. The number of the pregnancy is also wholly immaterial, different as are the causes alleged for its criminal induction, and equally liable in youth and age, as women seem to be, to accident or placental disease. Whitehead and West are of opinion that abortion naturally resulting is most common after the sixth pregnancy,* but the point needs further investigation.

Among the proofs of intent must be included, as we have seen, the excuses offered by the accused or suspected party, and the means resorted to for consummation. These we now proceed to examine.

It will be evident that the plea of necessity can be made by none but a medical man. We shall show that the cases where abortion is legitimated by the rules of science are extremely few, and that for safety's sake their applicability should in no instance be allowed to rest upon a single opinion.† For all others beside the physician there can be no allowable excuse except, in the mother's case, insanity; which, however common in the true puerperal state, and often no doubt then showing itself by infanticide, has in early pregnancy, and to any extent, still to be observed. Other pleas as offered by the mother, ignorance of pregnancy or of fœtal life, duress, personal health or that of her family, accident, carelessness, fear of child-bed, malpractice on part of the attendant, we have already considered at sufficient length. It is sometimes effected in hatred of the husband or in jealousy, sometimes for concealment of shame; excuses of little more value than those of extravagance or fashion. Constitutional predisposition can hardly be asserted, unless the miscarriage have been preceded by others; very many ineffectual attempts are on record, although the existence of such predisposition was evident. It will often be alleged that the measures instituted were to prevent instead of to effect the mis-

---

* Med. Times and Gazette, Jan., 1856, p. 611.

† "This operation must not on any account be undertaken without the sanction, and in the presence, of another practitioner."—CLAY, Hand-book of Obstetric Surgery, p. 13.

carriage, and that this has resulted in consequence merely of an excess of good-will; the sophistry is generally apparent.

The means resorted to are for two purposes: on the one hand, to prepare the patient for the abortion and preliminarily to lessen her danger, or to conceal the character of those, on the other hand, which really occasion it, and for this end used prior or subsequently to them. We may yet take occasion to consider these several agents in some detail; it remains only to remark that their use in any given case must be compared with what was then actually needed, or would have been required had the abortion been justifiable and necessary.

Certain drugs, ergot and savin for instance, the class of so-called abortives, popularly considered specific, are always suggestive of evil intent. They would not be used, were abortion necessary, by a well-informed practitioner, caring for the life of the parent or fœtus. The same is true, though of course to a more limited extent, of all over-drugging, over-manipulation, or over-exertion by a pregnant woman, by whomsoever advised or performed. In every instance it is necessary to compare the cause alleged with the effects observed, and to judge of it from these. Where direct operative manœuvres are suspected or charged, the processes or instruments, the results, immediate and consecutive as well as remote, the period elapsing before their occurrence, must all be taken into careful consideration.

But, on the other hand, it is immaterial what was the agent, and whether or not it would produce abortion, if it was believed capable of this effect, and employed or administered with that intent. If the person charged knew that the woman was with child, and the probable effect of the agent administered, this is good presumptive evidence that the intent was to produce the miscarriage, and where the effect of abortion is actually thus produced, it will materially aid the presumption of such intent.*

It was stated early in this inquiry that a difference existed between the methods of investigation, as regards the examination of the fœtus, proper in abortion and infanticide. The reason of this has been pointed out by Tardieu.† In the latter case, the whole matter turning upon the questions whether the child was born living or dead, and in which of these states it was injured, it becomes necessary to prove one or the other of the alternatives, but in abortion they are intrinsically of no importance whatever. The only points then to be decided are, was the birth premature, and if so, was it intentional, and if so, was it absolutely essential and to save either maternal or fœtal life. Except as bearing on these questions,

---

* 1 GABBETT, Cr. Law, 523. † Loc. cit.

therefore, it is of no consequence whether wounds were inflicted, whether the lungs had been inflated, whether the fœtus was viable, or even whether it was ever discovered.

In their place, however, these points are each important, but only as bearing on the main facts to be determined. In a case, for instance, as that related by Ollivier d'Angers, where the fœtus, though very immature, lives several hours after its expulsion, this fact alone will preclude the idea of a slow and progressively acting cause like most forms of abortive disease, and will point to some direct interference, by means suddenly terminating the pregnancy without injuring the fœtus.

And so in other cases, especially of sudden maternal death, it is of importance to ascertain as nearly as possible the period at which death of the fœtus took place. If the two were coincident, the deduction might be other than if the latter were proved to have preceded the former by several days. The differences observed between putrefaction and decomposition *in utero* and in the open air must not be lost sight of. In the one case, according to Orfila, Devergie and Martin, Moreau, P. Dubois, Danyau, Cazeaux, Tardieu, and my own experience, a uniform and characteristic reddish-brown hue obtains in proportion to the time of retention after death, varied perhaps by the action of the amniotic fluid;* the fœtus wrinkles, dries, and becomes mummified, unless in earliest pregnancy, when it generally resolves itself into a gelatinous mass.

If the cervix, the portion of the uterus most frequently wounded, is found punctured or lacerated, while the ovum is still retained, there is reason for suspicion; if the membranes are torn and extensively detached, while the cervix is but little dilated, such is increased; and it is made almost a certainty, if with the latter condition, nothing remain of the ovum in the uterine cavity but lacerated fragments. Here the abortion would probably not merely have been intentionally induced, but by the direct introduction and agency of instruments.

Wounds of the fœtus are much rarer than those of the mother, and are usually simple pricks of the skin marked by blackish coagula or extravasations; which, if upon the skull and unless care be used, are liable to be simulated by clots casually adhering to the hair or scalp. If the wound be deeper, its course may be traced by dissection. Its situation varies; Devergie thinking it always on the back and buttocks, while Tardieu, with some warmth, would restrict its location to the top of the cranium. This difference is easily explained by variations in the time of pregnancy, and in consequence partly, as may also depend on its life or death, in the presentation of the child. The rarity of their occurrence, though denied by

---

* CHEVALIER and DEVERGIE, Ann. d'Hyg., 1856, p. 157.

Taylor* and other medical jurists, might, however, be expected, and is readily accounted for; the instruments used by ignorant persons seldom entering the os, however severely wounding the cervix, and where they do enter, usually only piercing the membranes; against which, except toward the end of pregnancy, in a deficiency of liquor amnii, or in labor, the fœtus can hardly be said to forcibly press.

### 4. *The Identity of the Party Accused.*

Most of the points here involved having already been incidentally considered, we will not repeat them. The circumstances and history of the case, the relative correspondence of different testimony, the allegations of the accused, will all bear directly upon the question at issue.

In trials for abortion, of all others, the medical witness and the advocate should bear in mind their liability to error; the juror and the judge the fact that innocent persons are at times wrongly accused, often by the true criminals themselves.

In defence, it must either be pleaded that the alleged abortion did not occur, that it was accidental or natural, that it was necessitated, or that it was induced by another than the individual charged.

The first of these pleas is seldom offered except in the earliest months of pregnancy, and would be invalid if an attempt at the abortion could be proved. In default of this, however, where the ovum has been lost, or has passed unnoticed, the fact that the sanguineous effusion was hemorrhagic and attended with clots, would, in the absence of any uterine disease sufficient to account for this, be so far presumptive evidence; to be corroborated or not by the history of the case. Moles and hydatids are now generally allowed to be mere transformations of the product of conception; their premature discharge, therefore, equally an abortion.

In answer to the second plea, the importance of several points must be borne in mind. It has been well put by Tardieu that it is wrong to commence, as advised by most authorities on the subject, by enumerating all the natural and accidental causes liable to have produced the abortion. On the contrary, the signs and proofs of criminal violence should first be sought, and these compared with the allegations of witnesses and the possibility of a natural or accidental origin. The traces of falls, contusions, and wounds must be found, not believed on mere allegation; coincidences must be guarded against, equally with untruth.

We have already laid down rules here available; that the state of the fœtus often affords proof of the cause of its expulsion, this being slow

* Med. Jurisp.; Griffith's ed., p. 472, Hartshorne's ed., p. 378.

and natural, and depending on disease and predisposition, or not; that in flagrant malpractice, the use of alleged specifics, or of measures likely to produce direct miscarriage, or otherwise absolutely counter-indicated by the general health and constitution of the patient, a contradiction exists to the plea offered, in itself strong presumptive evidence of criminal intent; and that in certain cases this evidence becomes positive, as where, for instance, a sponge found or proved to have been inserted into the os uteri as a dilating tent, is alleged to have been intended as a mere pessary and placed in the vagina.

If the accused be a physician, presumed as he should be, acquainted with the great principles of practice, his only plea can be, where the means used were unjustifiable and proved such, and where the pregnancy was known to others, that he was ignorant of its existence. Liable as the profession are at any time to this charge, and easy as it is in almost every case, especially of instrumental procedure, for us to take such preliminary measures as would be likely to settle the question of the existence of pregnancy, or to request the presence of a witness to our act, it is unjust to ourselves and to each other to omit these precautions.

But if, on the other hand, the charge be utterly unfounded, it is probable, as I have already remarked, that contradictions in the testimony or the alleged facts could always be shown to exist, and the perjury thus exposed. It would be self-evident, were the accused proved to have been first consulted after the abortion had terminated, though not if it had only commenced.

### V. ITS PERPETRATORS.

It is interesting, and at the same time of judicial importance to ascertain, so far as possible, the standing and character of the perpetrators of this crime.

In the first place, French statistics on the large scale show that the number of criminals, principals and accomplices, in that country at least, is in large excess to the instances of the crime, there having been in 183 trials, from 1826 to 1853, not merely 417 parties accused, but 213 convicted; and that in 75 per cent. of the prosecutions and convictions occurring, where the abortion is not induced by the mother herself, the offenders are women.* With us the same statement is, without doubt, equally true.

The part played by the mother, herself so often a victim, is almost always that of a principal, yet as laws now stand, she can scarcely ever be reached. The cases where she is under duress, by threat of other per-

* TARDIEU, loc. cit., 1856, p. 124.

sonal violence from her husband or seducer, and thus compelled to submit to abortion, or where the act is performed by his direction but without her knowledge, are so rare, that in a general statement they may be assumed not to exist. If the mother does not herself induce the abortion, she seeks it, or aids it, or consents to it, and is, therefore, whether ever seeming justified or not, fully accountable as a principal. We have already seen the position these mothers hold in the community, high as well as low, rich as well as poor, intelligent and educated as well as ignorant, professedly religious as well as of easy belief, not single alone, but married.

We turn now to their partners in guilt, more criminal than themselves; for whatever excuse the latter may suppose themselves to possess, the former can have none.

The accomplices in criminal abortion are of several classes, distinguishable in some respects from each other, especially by the relative frequency with which their part is played. They are:

Of women, I.—Friends and acquaintance.
II.—Nurses.
III.—Midwives and female physicians.
Of men, IV.—Husbands.
V.—Quacks and professed abortionists.
VI.—Druggists.
VII.—And worst of all, though fortunately extremely rare, physicians in regular standing.

In many cases, such at least is judged by the writer from his own observation, the abortion is not merely advised, but induced by some female *friend*, especially by one who has herself undergone, in her own person, the crime; perhaps without appreciable evil result,—but this is not necessarily the case, for even where such result is present and plainly in consequence, its connection with the true cause is frequently unsuspected or disbelieved.

It has been said that misery loves companionship; this is nowhere more manifest than in the histories of criminal abortion. In more than one instance, from my own experience, has a lady of acknowledged respectability, who had herself suffered abortion, induced it upon several of her friends, thus perhaps endeavoring to persuade an uneasy conscience that by making an act common, it becomes right. Such ladies boast to each other of the impunity with which they have aborted, as they do of their expenditures, of their dress, of their success in society. There is a fashion in this, as in all other female customs, good and bad. The wretch whose account with the Almighty is heaviest with guilt, too often becomes a heroine. So true is the case, that the woman who dares at the present day publicly or privately to acknowledge it the holiest duty of her sex to bring forth living children, "that first, highest, and in earlier times almost universal lot,"* is worthy, and should receive, the highest admiration and praise.

The ease with which an accomplice is procured, provided the idea originates with the victim herself, and is not suggested by another, is found among *nurses* to be greatly increased. We separate them as a class from midwives and female physicians, with whom, though in this country not generally acknowledged or thought identical, they not unfrequently aim to be confounded. They are usually, and rightly, thought more familiar with the laws of health and disease, than the generality of their sex; they are, if doing their duty in his sight, seen to be treated with respect by the physician; they are commonly of mature age, supposed discreet, wise, and to keep their own counsel; they have had opportunities of gaining the confidence of the mother; many of them have themselves borne families. They are therefore approached with less hesitation, and are not always found proof against an offered fee.

What we have said of nurses applies with increased pertinency to *female physicians* and *midwives.* These make it their claim, in rivalry of the male physician, that their schools and their practice are, like his, founded on those abroad, especially of Paris. Tardieu shows, in a total of 32 cases occurring in that city and collected by himself, that in 21—no less than 66 per cent., or two-thirds of the whole number reported—the crime was perpetrated by midwives.† This class frequently cause abortion openly and without disguise. They claim a right to use instruments, and to decide on the necessity and consequent justifiability of any operation they may perform. Where they establish private hospitals, professedly for lying-in women or not, their chances, previously great, of committing this crime and infanticide with impunity, become more than doubled. It has been found necessary in France for the police to exercise rigid surveillance over these establishments. In one instance, occurring at Grenoble, it was proved that within three years there had happened in the house thirty-one still-births at the full time, or deaths just after birth, and that the abortions and miscarriages had been almost innumerable.‡ In another case, to conceal the evidence of these truly *corpora delicti,* and to evade the law against secret burials, the midwife had established an understanding and a current account with an undertaker, who was accustomed to smuggle her fœtuses into his coffins, by the side of the corpses confided to him for burial. In still other cases, the victims are kept on

* A Woman's Thoughts about Women. By the author of "John Halifax, Gentleman." 1858, p. 14.

† Loc. cit. ‡ Ibid.

hand, preserved in jars; private collections vieing in extent with those of legalized obstetric museums.

By these remarks we would not be supposed endeavoring to excite prejudice against female physicians and midwives, as such, or advocating their suppression. We are now merely considering this crime of abortion, in relation to which they are peculiarly and unfortunately situated. At present everything favors their committing the crime; their relations to women at large, their immunities in practice, the profit of this trade, the difficulty, especially from the fact that they are women, of insuring their conviction. Let better laws be enforced, and let public opinion be enlightened concerning the guilt of abortion, and the influence for evil of this class of offenders will in great measure be done away with.

Of male abortionists we have less to say. Their number is fewer abroad, bearing the proportion, as we have seen, of but one to every four, and their liability of being applied to, or consulted, is slight in comparison.

*Husbands*, though generally knowing to the offence of their wives, and often counselling it, probably but seldom attempt its commission themselves; yet instances of this do undoubtedly occur. In but a sixteenth of the cases reported by Tardieu, was any compulsory violence exerted over women by their husbands.

*Druggists* and *professed abortionists* are accountable for the greater number of the cases of the crime attributable to men. The latter class, though proportionally rare, yet abound in every city, and take all means of making themselves known. A knowledge of their alleged specifics, against the use of which, "at certain times," the public are "earnestly cautioned," etc. etc., is brought home to all our women, no matter how purely minded, and despite every care to the contrary, through the medium of the daily press; few papers, however professedly respectable or religious, proving able to refuse the bribe.

Druggists, as a class, are little more than the confessed agents of these villains. Even should they not directly recommend their nostrums, as, however, is frequently the case, they almost universally keep them on sale, labelled to catch the eye, and placarded on their walls. Like the publishers and vendors of obscene literature, they conceive they are not to blame for supplying a public demand, however much they themselves may have done toward its creation.

And in this connection we must again allude to the guilt of the public press, which has proved itself so constantly and so dangerously an accessory to the crime. It would be thought that in Massachusetts, for instance, a statute like the following might do something to check this license:*

---

* Mass. Laws of 1847, chap. 83.

"Every person who shall knowingly advertise, print, publish, distribute, or circulate, or knowingly cause to be advertised, printed, published, distributed, or circulated, any pamphlet, printed paper, book, newspaper, notice, advertisement, or reference, containing words or language, giving or conveying any notice, hint, or reference to any person, or to the name of any person, real or fictitious, from whom, or to any place, house, shop, or office where any poison, drug, mixture, preparation, medicine or noxious thing, or any instrument or means whatever, or any advice, directions, information, or knowledge, may be obtained for the purpose of causing or procuring the miscarriage of any woman pregnant with child, shall be punished," etc. etc.*

The above statute, however, such is the public sentiment on this point, is not enforced, or is daily evaded. The press, if it choose, may almost annihilate the crime; it now openly encourages it.

It has been often alleged, and oftener supposed, that *physicians in good standing* not unfrequently and without lawful justification, induce criminal abortion. This statement, whatever exceptional cases may exist, is wickedly false. The pledge against abortion, to the observance of which Hippocrates compelled his followers by oath,† has ever been considered binding, even more strongly of late centuries. The crime is recognized as such in almost every code of medical ethics; its known commission has always been followed by ignominious expulsion from medical fellowships and fraternity. If this direct penalty be at any time escaped, it is only through lack of decisive proof, bare suspicion even of the crime insuring an actual sundering of all existing professional friendships and ties; a loss that subsequent proof of innocence could hardly restore. Such is the unanimous feeling of the profession; to its credit be it said, that with but a single exception,‡ and this to his eternal disgrace, its writers are all agreed, abstractly considering the subject, on the sanctity of fœtal life. The instances where physicians in good standing are guilty of the crime, are of rare occurrence; the error that has prevailed on this point, originating from the self-assumed titles of notorious quacks and knaves. But no condemnation can be too strong for the physician who has thus forgotten his honor; who has used to destroy life, that sacred knowledge by which he was pledged to preserve it.

The criminal abuses likely to arise from the procurement of justifiable

---

* "By imprisonment in the State prison, house of correction, or common jail, not more than three years, or by fine not exceeding one thousand dollars."

† Opera omnia. Ed. 1655, i., p. 643.

‡ JÖRG of Leipsic, who speaks of the human fœtus as "only a higher species of intestinal worm, not endowed with a human soul, nor entitled to human attributes."

abortion by medical men, are so numerous, their own liability to be thought by the public criminally careless of fœtal life, or skeptical concerning its existence, is so great, that the subject is worthy special consideration. This I shall now devote to it.

(TO BE CONTINUED.)

